# Eyes wide open: Regulation of arousal by temporal expectations

**DOI:** 10.1016/j.cognition.2022.105062

**Published:** 2022-07

**Authors:** Nir Shalev, Anna C. Nobre

**Affiliations:** a Department of Experimental Psychology, University of Oxford, UK; b Oxford Centre for Human Brain Activity, Wellcome Centre for Integrative, Neuroimaging, Department of Psychiatry, University of Oxford, UK

**Keywords:** Arousal, Sustained attention, Temporal expectations, Theory of visual attention, Pupillometry

## Abstract

Maintaining adequate levels of arousal is essential for sustaining performance on extended tasks. To investigate arousal in prolonged tasks such as driving studies have traditionally used monotonous task designs. Both ecological and experimental settings often contain embedded temporal regularities, but it is unknown whether these enable adaptive modulation of arousal. We explored whether temporal predictability can modulate arousal according to the timing of anticipated relevant events. In two experiments, we manipulated the temporal predictability of events to test for behavioural benefits and arousal modulation, using pupillometry as a proxy measure. High temporal predictability significantly lowered the tonic level of arousal briefly increased arousal in anticipation of upcoming stimuli, whereas low temporal predictability resulted in tonically elevated arousal. These novel findings suggest that arousal levels flexibly adapt to the temporal structures of events and bring about energy efficiencies in the context of high levels of behavioural performance.

## 1 Introduction

The ability to anticipate the timing of an upcoming event allows us to prepare and perform more efficiently (Nobre & van Ede, 2018). In experimental settings, temporal expectations are typically manipulated on a trial-by-trial basis, to estimate the behavioural benefits conferred by temporally informative cues (Coull & Nobre, 1998), short rhythmic stimulations (Jones, 2010), learned temporal probabilities (Ghose & Maunsell, 2002), or sequential effects (Capizzi, Correa, Wojtowicz, & Rafal, 2015; Los, 2010). These experimental approaches are highly relevant when trying to map the mechanisms that support temporal anticipation of discrete cognitive events: individual trials are clearly marked by a either a single cue or a series of events, followed by an anticipation interval, a unique response-relevant stimulus, and a response interval that is terminated when a response is recorded. Much less is known about how anticipation of events unfolds when we engage continuously with extended cognitive tasks, where there are no clear boundaries between individual trials*.* Arguably, the transition from discrete task events toward continuous task performance is an important step toward understanding human behaviour in natural settings. Many forms of human behaviour require sustained focus and repetitive patterns, such as driving home from work or sowing a quilt blanket or monitoring a data stream during an experimental session for occasional artifacts. In such contexts, behaviour relies heavily on the capacity to maintain an adequate state of arousal over an extended period of time, and is not fully captured by mechanisms restricted to the momentary engagement with perceptual events (Shalev, Bauer, & Nobre, 2019).

Experimentally, sustained performance is typically studied using Continuous-Performance Tasks (CPT). CPT designs all share the same basic characteristic of presenting stimuli in a continuous stream, irrespective of whether participants provide a response. In fact, in CPT designs, response windows are not marked at all: they occur in between the succession of stimulus appearance. This nuanced feature makes distinguish them from traditional cognitive experiments, with multiple, well-defined “task events” such as a cue, followed by an interval, a target, a response time window, feedback (sometimes), etc. Most CPT studies have emphasised the role of slow modulations in sustained attention and arousal functions as the key factor in performance (Davies & Parasuraman, 1982; Robertson & O'Connell, 2010; Sarter, Givens, & Bruno, 2001). The modulation of arousal is typically attributed to the passive, gradual habituation related to repetitive stimulation (Fortenbaugh, DeGutis, & Esterman, 2017; Mackworth, 1968; Parasuraman, Warm, & Dember, 1987). However, it is unknown whether briefer, embedded task-dependent regularities affording selective temporal expectations can also affect arousal.

In CPT, the temporal structure is typically defined by the intervals between successive perceptual events, irrespective of motor responses. For example, imagine yourself practising your tennis skills using a tennis-ball machine. The machine continuously shoots tennis balls at a certain pace, regardless of whether you managed to hit a ball. The machine may shoot balls at regular or irregular intervals. In principle, if the observer is sensitive to the temporal structure of events, then two factors likely influence performance: sustaining an appropriate state of arousal and selectively deploying temporal attention to an imperative stimulus (i.e., the tennis ball). In practice, little is known about the potential of such rhythm-induced selective temporal anticipation to punctuate the overall background state of arousal.

Hebb (1955) discussed arousal as a non-specific cognitive resource that “in effect makes organised cortical activity possible”. The effect of arousal on performance is characterised by an inverted U-shaped curve: with performance best at intermediate arousal levels and falling off progressively toward the two extremes of sleep (low arousal) and anxiety (high arousal) (Aston-Jones & Cohen, 2005; Hebb, 1955). Neural control of arousal occurs through noradrenergic modulation arising in locus coeruleus of the brainstem reticular activating system (Steriade, 1996), which in turn is regulated by cortical feedback (Aston-Jones & Cohen, 2005). In humans and in non-human primates, changes to pupil size reflect firing rate at the locus coeruleus-noradrenergic system and associated arousal levels (Joshi & Gold, 2020; Joshi, Li, Kalwani, & Gold, 2016; Rajkowski, 1993). Thus, under the right control conditions, pupil size provides a convenient, non-invasive, and well-validated continuous proxy measure for the arousal-related system in both human and animal models.

Arousal is often discussed in the context of continuous vigilance tasks (Greene, Bellgrove, Gill, & Robertson, 2009; Mackworth, 1968) but adaptation according to stimulus predictability in such contexts has not been investigated. The ability of predictable temporal structures in extended tasks to drive transient fluctuations in arousal is particularly important given wide utilisation of CPT tasks to characterise sustained attention in clinical populations (Lee & Park, 2006; Richards, Samuels, Turnure, & Ysseldyke, 1990; Shalev et al., 2019; Shalev, Humphreys, & Demeyere, 2016; Sims & Lonigan, 2012; Tsal, Shalev, & Mevorach, 2005). The possible contribution of selective temporal expectations to performance during CPT was suggested by a recent study in which temporal predictability was manipulated during a CPT (Dankner, Shalev, Carrasco, & Yuval-Greenberg, 2017). Individuals monitored a stimulus stream in a continuous task and responded to occasional pre-defined targets. The results showed that when targets appeared in a fixed rhythm, participants were faster and were more likely to inhibit their eye movements before stimulus onset. To our knowledge, we present the first evidence for perceptual benefits of temporal expectation in CPT while keeping motor demands at minimum (e.g., not limiting the response time window, emphasising accuracy over speed, and measuring performance only based on perceptual parameters). In our current study, we take the next important step and investigate whether performance benefits of temporal expectations within a CPT are associated with modulation of arousal levels.

Valid temporal expectations about the onsets of events in CPT can be a strong source of uncertainty reduction. Studies investigating the effects of uncertainty about stimulus identity or reward associations in the context or reinforcement learning have shown strong modulation of pupil size attributed to changes in arousal (e.g., (Friedman, Hakerem, Sutton, & Fleiss, 1973; Lavín, San Martín, Rosales Jubal, Martín, & Jubal, 2014; Preuschoff, 't Hart, Einhäuser, & Einhauser, 2011; Urai, Braun, & Donner, 2017; Vincent, Parr, Benrimoh, & Friston, 2019). Accordingly, we hypothesised that reduction of *temporal* uncertainty, arising from predictable rhythmic stimulus presentation within the context of CPT, would also lead to changes in pupil size. Whereas previous studies with human participants have confirmed that pupil measures track expected reward gains to individual stimuli in decision-making tasks (Lavín, San Martín, & Rosales Jubal, 2014; Preuschoff, 't Hart, & Einhauser, 2011), no study to our knowledge has investigated whether temporal predictions can modulate arousal modes within continuous-performance tasks. We hypothesised that increased temporal uncertainty would be associated with higher levels of tonic arousal, and that the availability of temporal regularities would enable a more efficient management of arousal. In line with previous studies, changes in arousal will reflect in pupil dilation and contraction.

Any CPT design inherently contains task-relevant, predictive temporal structures that can, in principle, aid performance. Even when the intervals between task-relevant stimuli vary, informative temporal structures come about through learned associations based on the trial history of temporal intervals or the cumulative conditional probability of events occurring given the span of intervals (Los, Kruijne, & Meeter, 2017; Nobre, Correa, & Coull, 2007). In addition, many CPT tasks use constant intervals and thereby provide a deterministic rhythmic structure that could support significant reduction of tonic arousal by strong temporal expectation.

Across two experiments, we measured perceptual discrimination and pupil size during continuous-performance tasks to test whether temporal expectations improve behaviour and modulate arousal. We used visual tasks and therefore chose an index of arousal (pupillometry) that was directly linked to the sensory modality most relevant to behaviour. The two experiments used slightly different designs but asked the same questions, thereby ascertaining whether the findings were reproducible and generalisable. In both experiments, we manipulated whether target events occurred in a temporally predictable or unpredictable fashion. Behavioural measures of perceptual discrimination tested whether performance improved when target onsets were fully predictable. Pupil traces probed whether the overall level of temporal expectations modulated the tonic arousal tone, and tested whether local changes in temporal expectation, resulting by rhythmic and probabilistic structures within the task were linked to short bursts of arousal related to stimulus anticipation.

## 2 Experiment 1

### 2.1 Methods

The experimental procedure was reviewed and approved by the central university research ethics committee of the University of Oxford.

#### 2.1.1 Participants

Thirty neurotypical young adults participated in this study (20 of whom were female, mean age 25, SD = 3.2). The sample size was chosen based on previous and ongoing studies using the same behavioural manipulation (Shalev et al., 2016; Shalev et al., 2019; Shalev, Humphreys, & Demeyere, 2018). Pilot data contrasting pupil size in a comparable continuous context (variable and fixed rhythms) have yielded medium to large effect sizes (Cohen's d values ranging between 0.5 and 0.7) using comparable samples (ranging between 25 and 30 participants per experimental group.) Power was calculated based on an effect size of 0.6 and a two-tailed significance level of 0.05 to yield a Power (1-β err probability) of 0.8, and resulted with a minimum sample size of *N* = 24. Participants were recruited through an online research-participation system at the University of Oxford. All were right-handed and had normal or corrected eyesight (based on self-reports). They were compensated for their time (£10 per hour).

#### 2.1.2 Apparatus

A PC with an i7 processor and a 2-GB video card was used for displaying stimuli and recording behavioural data. The task was generated using Presentation software (Neurobehavioural Systems, Albany, CA). The stimuli were presented on a 24” LED monitor, with a screen resolution of 1080 × 1920 and a refresh rate of 100 Hz. All stimuli were preloaded to memory using the presentation software to minimise temporal variability in stimulus display.

#### 2.1.3 Experimental design

**Fig. 1:**
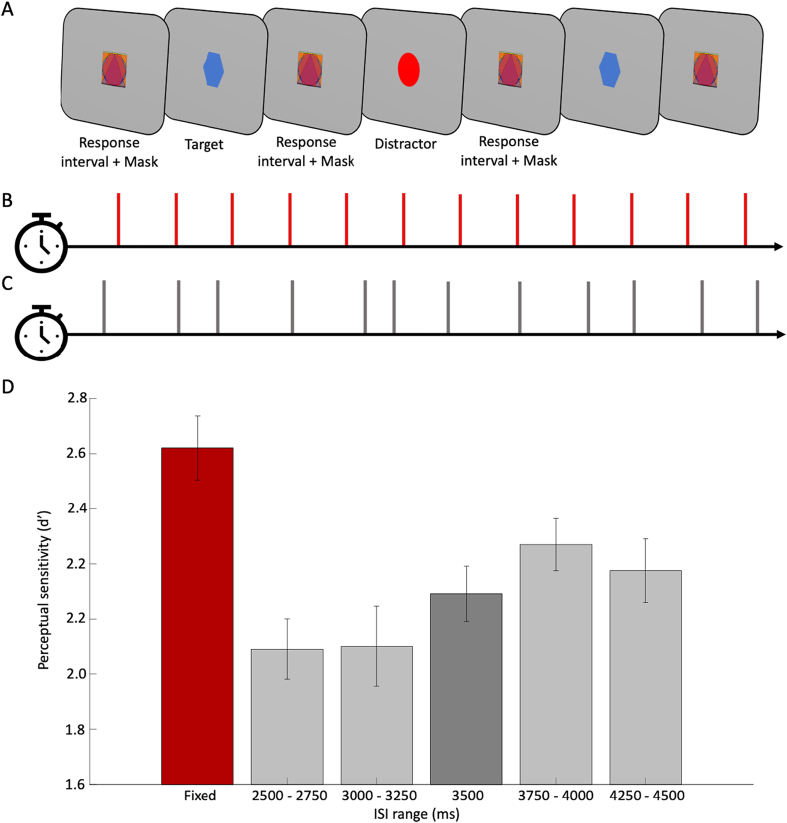
(A) a schematic illustration of the different conditions in the CPT. Participants had to respond when identifying a target (blue hexagon, designated as a ‘target’) and ignore all other shapes (for example a red circle, designated as a ‘distractor’). The stimuli – targets and distractors – were masked during the interstimulus intervals (‘mask’). Targets and distractors were presented in two conditions (blocked), which repeated two times within each experimental run. They were either presented at (B) fixed, 3500-ms intervals, or (C) at varied intervals between 2500 and 4500 ms in steps of 250 ms. In (D) we compare the perceptual sensitivity to targets appearing after 3500 ms intervals in the two experimental conditions. The red bar represents the mean perceptual sensitivity in Fixed blocks, during which all intervals were equal to 3500 ms. The grey bars depict the mean d′ on different subsets trials in variable blocks, based on the inter-stimulus intervals. Error bars represent the standard error of the mean.

Participants were requested to respond to target shapes and to ignore distractor shapes (see Fig. 1 for a schematic illustration). Targets and distractors briefly (120-ms duration) replaced a multi-shape masking stimulus (mask) that was otherwise continuously present. The masked version of the CPT was originally designed to reveal individual differences using perceptual parameters and has been validated in various populations (Shalev, Humphreys and Demeyere, 2016, Shalev, Humphreys and Demeyere, 2018; Shalev, Vangkilde, et al., 2019). Uniquely, in the current study the temporal regularity with which target and distractor shapes appeared was manipulated across conditions.

The mask comprised four superimposed figures (square, triangle, circle and hexagon) in different colours (blue, red, and green). Its total size was 5 degrees of visual angle, both horizontally and vertically. The mask appeared and remained at the centre of the screen, disappearing only when replaced by either a target or a distractor shape for 120 ms. The mask then reappeared immediately, generating pre- and post-masking of each target or distractor. The target shape was a blue hexagon, and distractor stimuli were either similar in colour to the target (blue circle or diamond), similar in shape (red or green hexagon), or completely different (green circle and red diamond). All distractor types appeared with an equal distribution. Two-thirds of events were targets and the rest were distractors. Distractors and targets appeared at the centre of the screen and occupied a square of 5 degrees of visual angle vertically and horizontally. Participants were instructed to press the ‘space bar’ whenever they detected targets, while ignoring and refraining from responding to distractors, in a continuous stream of stimuli lasting approximately 12 min (altogether, 133 target and 67 distractor stimuli appeared in between mask stimuli).

The interstimulus intervals (ISIs) between critical stimulus events (targets and distractors) were manipulated to vary the degree of rhythmic temporal expectation. There were four concatenated blocks of trials (not explicitly signalled) alternating between Fixed and Variable conditions. During Fixed blocks, targets and distractors appeared predictably every 3.5 s, in a rhythmic fashion. During Variable blocks, targets and distractors appeared unpredictably at intervals between 2.5 and 4.5 s (mean 3.5 s) with an equal probability for each ISI (drawn from 9 possible intervals, equally spaced between 2500 ms and 4500 ms in gaps of 250 ms). Fixed and Variable blocks were always interleaved, and their order was randomised among participants.

#### 2.1.4 Procedure

The experiment took place in a dark testing room. Participants sat 50 cm from the monitor, and a chin rest was used to keep their head still. The experimenter was also sitting in the room, behind the participant, to monitor behaviour. The task instructions appeared on the screen and were explained to the participant by the experimenter, who also answered any questions about the procedure. The session lasted approximately 12 min, and commenced with a short practice session of 10 trials.

#### 2.1.5 Statistical analysis

##### 2.1.5.1 Behavioural benefit

First, we sought to verify whether individuals utilised rhythmic structures to benefit behaviour (as previously observed by Dankner et al., 2017). Responses were categorised as being: a correct response (responding to target), a correct rejection (withholding response to distractor), a false alarm (responding to distractor), or an omission (not responding to target). We derived perceptual parameters based on the Signal Detection Theory (SDT; Green & Swets, 1966) to estimate the perceptual sensitivity (d′) and response bias (‘criterion’; β). To correct for extreme cases where performance reached ceiling, we applied a loglinear correction (Hautus, 1995).

First, we focused only on perceptual judgments that followed a 3.5 s interval, which was used in the isochronous rhythmic blocks and preceded 20% of the trials in the Variable condition, since this allowed us to learn whether responses to targets were affected by the fixed/variable contexts, while controlling for other temporal factors that may influence performance, such as passage of time or hazard rates (Nobre & Rohenkohl, 2014). We used a *t*-test for this analysis. Subsequently, to estimate the contribution of passage of time and hazard rates to performance, we calculated the perceptual sensitivity on five ranges of intervals between stimuli: trials that followed 2500–2750 ms intervals; 3000–3250 ms; 3500 ms; 3750–4000 ms; 4250–4500 ms. We used an ANOVA test to evaluate a linear contrast, as an indication for an increase in perceptual performance as a function of the interval range. A t-test and ANOVA procedures were selected as they are most suitable for modelling normally-distributed perceptual parameters that were calculated across trials.

##### 2.1.5.2 Pupillometry

Pupil size was recorded using a 2000-Hz sampling rate (1000 Hz per pupil), allowing the construction of time series. The analysis procedure first focused on overall differences in pupil size between conditions to identify tonic effects related to temporal expectations (*Tonic Effects* – a further description appears below). Consistent with the task parameters, the optimal time frame for identifying tonic effects within a trial was the period of 2.5 s following stimulus onset (2.5 s being the shortest ISI and therefore allowing the inclusion of all trials). In addition, we estimated tonic effects by averaging pupil size during entire blocks (50 trials, over 3 min). Then, to identify markers of pupil changes due to temporal expectation, we analysed the changes in pupil size during the 1-s period preceding stimulus onset (*Anticipation* – a further description appears below). The raw pupil data were converted to a data matrix using a MATLAB script, excluding data from practice and breaks between blocks. Blinks were interpolated using a cubic spline interpolation (Mathôt et al., 2013), using a dedicated Matlab function available as part of the Pupil Response Estimation Toolbox (PRET) (Denison, Parker, & Carrasco, 2019). Data were smoothed using a moving average widow, based on the ‘smoothdata’ MATLAB function. This method computes a window size based on a heuristic set to attenuate approximately 10% of the energy of the input data (smoothing factor set to 0.1).

Tonic differences in arousal between conditions were calculated based on a direct comparison of standardised pupil size. The interpolated and smoothed data were first converted to z scores, by calculating for each participant separately the mean and standard deviation of pupil size throughout the entire experiment and converting all the pupil samples to z scores. We then calculated the mean standardised pupil time-series for each participant, on each block type, which resulted in a time series extending over approximately 3 min, consisting ~180,000 samples (sampling rate in milliseconds). We then compared the overall mean standardised pupil size for each participant on each condition using a repeated-measures permutation *t*-test (based on 50,000 repeated samples).

To look at trial-level modulation of pupil diameter, we constructed two time-series of different lengths: 3500 ms in Fixed blocks, which was the same interval throughout the experiment; and 4500 ms, which was the maximum length of a single trial in Variable blocks. After the minimal inter-stimulus interval in Variable blocks (2500 ms), time series include a varying and decreasing number of observations until the maximum interval of 4500 ms. We contrasted the two time-series directly with a permutation t-test based on 50,000 samples. The resulting distribution for each data point (each millisecond of recorded data) was compared to a critical t-value (*p* < .05) adjusted for multiple comparisons based on the ‘t-max’ method (Blair & Karniski, 1993). This approach is unaffected by autocorrelations among successive measurements and was used by previous studies to assess changes in pupil diameter over time (e.g., Zokaei, Board, Manohar, & Nobre, 2019). We focused on a time window between 1000 ms and 2500 ms after the onset of the visual mask which immediately followed a target or distractor stimuli. This time window was chosen to reduce the influence of the transient pupil responses caused by a change in visual stimulation and matches previous work in our lab (e.g., Zokaei et al., 2019) as well as our pilot data (see Shalev, 2017; Shalev, Demeyere, & Nobre, 2017; Shalev & Nobre, 2018).

Next, we tested for effects of temporal preparation or anticipation based on the passage of time and mounting conditional probabilities at the single-trial level. We focused on the time series in Variable blocks, which included multiple interstimulus intervals. We used a linear regression to evaluate the slope of change in pupil size between 2000 ms after onset until 4500 ms, based on the mean pupil time-series we constructed for each participant. The data were then fitted and compared to a constant model. Additionally, to estimate the relationship between hazard rate and pupil size directly, we calculated the objective Cumulative Distribution Function (CDF) of the interstimulus intervals. We then computed the Pearson's correlation between the pupil-size time series and the CDF.

Finally, we examined the three-way relationships among temporal structure, pupillometry, and behaviour. To measure performance, we used accuracy. The model included the temporal regularities (Fixed / Variable) as a predictor. Accuracy was modelled as a function of the pupil size during stimulus onset (over 10 ms, the equivalent of a single refresh rate of the monitor). To allow direct comparisons across conditions, we only included trials that followed 3500 ms interstimulus interval.

Raw pupil size was standardised for each participant, i.e., we first calculated the mean and standard deviation for each participant across all trials and then converted the pupil size to a z-score. Three factors were included as predictors in a logistic regression (using a binomial generalised linear model): Standardised pupil size (first and second order polynomial), temporal regularities (fixed vs variable), and ISI. The inclusion of first and second order polynomial fits was intended to account for the non-linear arousal function (Aston-Jones & Cohen, 2005). The final regression equation included the additive effect of pupil size, temporal regularities, and the interaction between pupil size and temporal regularities. The model was compared to a null model using a chi-squared test.

##### 2.1.5.3 Data exclusion

Before running statistical comparisons, we evaluated the number of usable trials for each participant. Usable trials were those in which there was a valid record of the pupil in at least one eye (left or right), and in which eyes were fixated at the centre of the screen. As a default, we focused on analysing the data from the right eye. We identified five participants who had a substantial number of missing trials (50% and more), and they were excluded from the study prior to any further analysis. The final set of analyses included 25 participants.

### 2.2 Results

#### 2.2.1 Behavioural data

Our results indicated that participants were better at detecting a target after 3500 ms when it appeared within a Fixed block, compared to when it appeared after the same interval in a Variable block (t(24) = 4.05; *p* < .001 95%CI = [0.259;. 798]). There were no differences in the response bias (β) (*p* = .455). Descriptive data across intervals and conditions appear in Table 1. An illustration of the task, along with the perceptual sensitivity for targets in the two conditions, appears in Fig. 1.

**Table 1:** Descriptive statistics. Mean and standard errors (in parenthesis) of perceptual sensitivity (d′) and response bias (β) at different intervals and conditions. The left column represents the means during Fixed blocks, when all intervals were 3500 ms. The other five columns show the average perceptual parameters in Variable blocks split by interval range: when intervals were 1) 2500 and 2750 ms, 2) 3000 and 3250 ms, 3) 3500 ms, 4) 4000 and 4250 ms, and 5) 4250 and 4500 ms.

	Fixed	Variable				
	3500 ms	2500–2750	3000–3250	3500	3750–4000	4250–4500 ms
d′	2.82 (0.11)	2.09 (0.11)	2.10 (0.14)	2.29 (0.10)	2.46 (0.09)	2.37 (0.11)
β	−0.16 (0.08)	0.03 (0.06)	−0.08 (0.07)	−0.11 (0.06)	−0.07 (0.06)	−0.09 (0.07)

In addition to the performance benefit in the fixed-interval condition, the data in Fig. 1 revealed a pattern of increase in perceptual sensitivity as a function of the inter-stimulus interval in the variable-interval condition. To evaluate this pattern statistically, we used an ANOVA to test for a linear contrast. The dependant variable was the mean perceptual sensitivity, and the interval range (2500–2750 / 3000–3250 / 3500 / 3750–4000 / 4250–4500) was the independent factor. We included only trials in Variable blocks. The result indicated a significant linear trend (F(1,24) = 9.668; *p* = .005; partial η^2^ = 0.287). Such a pattern is in line with previous reports of the increase in the conditional probability for targets to appear as a source that drives temporal anticipation (‘hazard rates’) (Correa & Nobre, 2008; Ghose & Maunsell, 2002; Janssen & Shadlen, 2005), however in our task this could not strictly be separated from putative effects of general mounting preparation over time (Coull, 2014; Nobre, 2010).

#### 2.2.2 Pupillometry data

**Fig. 2:**
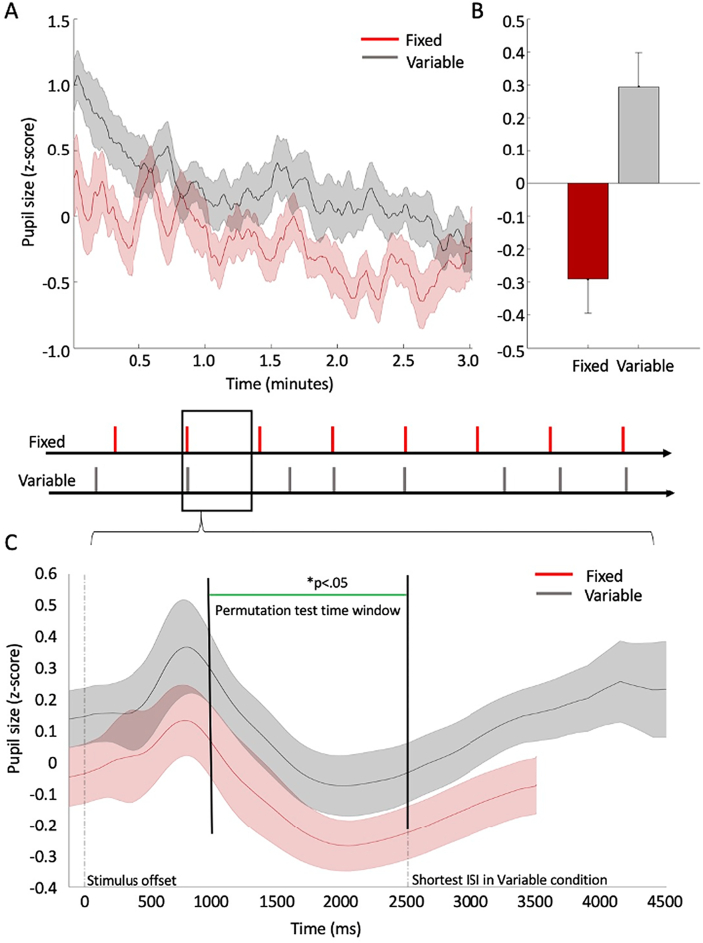
A) average pupil size (standardised) in Fixed blocks (in red) and Variable blocks (in grey) plotted across all participants, over the duration of experimental blocks, lasting approximately 3 min. Lines represent the mean and shaded error bars represent standard error. For illustration purposes only, data were smoothed using a time window of 7000 ms (equivalent to an average duration of two trials) and the data were standardised by converting the raw pupil size to z scores. The conversion was done separately for each participant, based on the mean pupil size (and standard deviation) for that individual throughout the entire experimental run (irrespective of experimental condition). (B) contrasting the mean pupil size (standardised z-scores) during Fixed blocks (red bars) vs. Variable blocks (grey bars). Error bars represent standard error. (C) (Standardised) Pupil size over the duration of individual experimental trials averaged across all participants in Fixed blocks (in red) and Variable blocks (in grey). Lines represent the mean and shaded error bars represent the 95% confidence interval range. All trials in Fixed blocks lasted 3500 ms. In Variable blocks, trial duration varied between 2500 and 4500 ms. Therefore, the grey time series includes a variable and decreasing number of observations within the range of 2500–4500 ms following stimulus offset. For illustration purposes only, data were smoothed using a time window of 250 ms (equivalent to the gap between two adjacent interstimulus intervals in the Variable condition). The data were standardised by converting the raw pupil size to z scores. The conversion was done separately for each participant, based on the mean pupil size (and standard deviation) throughout the entire experimental run (irrespective of experimental condition). We used a permutation *t*-test to compare the time-series within the range of 1000 to 2500 ms following stimulus offset. The permutation t-test was based on 50,000 samples. The resulting distribution for each data point (each millisecond of recorded data) was compared to a critical t-value (*p* < .05) adjusted for multiple comparisons based on the ‘t-max’ method (Blair & Karniski, 1993). The green line indicates the time points at which the two time-series differed significantly. (For interpretation of the references to colour in this figure legend, the reader is referred to the web version of this article.)

Our first analysis of the pupillometry data compared tonic differences in arousal level across all trials in a block, as a marker of adapting arousal to the temporal properties of the task. We contrasted the mean pupil size over the full duration of Fixed vs. Variable blocks. Fig. 2a shows the average standardised pupil size separated by block type. The two standardised signals were compared using a permutation *t*-test with the mean standardised pupil size in each condition as the dependent variable (see Fig. 2b). There was a significant difference (t(24) = −2.88; *p* = .008; 95%CI[−1.00; −0.16]), with pupil size significantly larger over the Variable block compared to the Fixed block.

Our second set of analyses characterised anticipation-related short-term changes in pupil diameter. We constructed a time series of the standardised pupil size immediately after stimulus offset and throughout a trial until the onset of the subsequent stimulus, to observe event-locked changes in pupil size. Fig. 2c shows the contrast between the mean pupil size in Fixed blocks and in Variable blocks. After the minimal inter-stimulus (2500 ms), the time series in Variable blocks include a varying and decreasing number of observations until the maximum interval of 4500 ms.

We used the stimulus-locked time series to retest for tonic differences, this time at the trial level. Permutation *t*-tests comparing smoothed and interpolated pupil size after the initial stimulus-evoked response (1000 to 2500 ms) showed a consistent difference between the conditions throughout the time window of interest as indicated by the green line in Fig. 2c. The permutation test was based on 50,000 iterations and was corrected for multiple comparisons based on the ‘t-max’ method (Blair & Karniski, 1993) (see Methods section).

As seen in Fig. 2c, after the initial stimulus-related pupillary response, a similar gradual increase in pupil size occurred in both Fixed and Variable conditions, commencing approximately 2000 ms after stimulus onset time. In the Variable condition, the increase in pupil size followed the increasing interval and thus the increasing probability for stimulus presentation. To characterise this trend statistically, we modelled the data using a linear regression. First, for each participant, we calculated the mean time series of pupil size between 2000 ms after onset, until 4500 ms. The data were then fitted and compared to a constant model. The result indicated a significant slope (R^2^ = 0.158; F(1,62,498) = 11,767; *p* < .001; partial η^2^ = 0.158). We also calculated the linear correlation between the time series of pupil size and the Cumulative Distribution Function of the interstimulus intervals based on the experimental data. This procedure allowed us to test the relationship between hazard rate and pupil size directly. The results indicated a significant correlation with a medium effect size (r(62498) = 0.41; *p* < .001).

Finally, we modelled accuracy data using a single model accounting for pupil size and temporal regularities. There was a significant, negative linear association between the linearly fitted standardised pupil size and accuracy (β = −10.84; SE = 3.94; z = −2.75; *p* = .005) and a significant interaction between temporal regularity (fixed vs variable) and the linearly fitted pupil size (β = 18.36; SE = 7.20; z = 2.55; *p* = .01). There were no other significant effects (all p's > 0.15). To follow up on the significant interaction, we remodelled the pupil size separately for each temporal regularity (fixed vs variable). There was a significant, negative association between performance and pupil size in Fixed blocks (β = −10.61; SE = 3.71; z = −2.85; *p* = .004) but no significant relation in the Variable blocks (β = 6.21; *p* = .118).

## 3 Interim discussion

When presented with a fixed rhythmic structure in a continuous task, individuals flexibly adapted their arousal level. The benefit of expectations was reflected in the behavioural data and was associated with a decrease in tonic arousal. Tonic differences were noted when comparing pupil size over the entire duration of blocks with and without rhythmic temporal structure. When comparing epochs around each stimulus, reliable tonic changes were observed within the time window between 1 and 2.5 s after the stimulus, after the pupillary reaction to the visual stimulus had settled and no visually induced changes occurred.

Rhythmic stimulus presentation in the Fixed condition afforded strong temporal expectation, as reflected in behavioural performance. However, a degree of temporal anticipation also occurred in the variable condition, as the interval preceding stimulus appearance increased over time, starting from 2500 ms (hazard rate). Thus, multiple temporal structures embedded in the CPT influenced behaviour: rhythm-based predictability in the Fixed condition, and the mounting probability of stimulus appearance over time in both the Variable condition. Notably, hazard rates may influence short-term pupil dynamics in the Variable condition, leading to a phasic ramping of the pupil size in anticipation of stimulus appearance – reflected by a linear increase in pupil size starting from ~2000 ms.

After establishing links between temporal structures and performance, and temporal structures and pupil size, we used a regression model to predict performance using pupil size and rhythmic structures combined (including only 3500 ms intervals). We found a significant negative association between pupil size and performance when modelling accuracy as a function of pupil size and block type (fixed vs. variable). This association interacted with the block-type factor and was found only in fixed condition. Therefore, when events appeared predictably, smaller pupil sizes were associated with better performance. This pattern is largely in line with our data indicating better performance in the fixed condition, in which the overall pupil size was smaller, compared to the variable condition. Notably, there was no association between performance and the block type (variable vs fixed). However, such result is not unlikely when accounting for pupil size – which was strongly affected by the block type manipulation, and therefore may account for the same variability in our data.

To test for the reliability and generalisability of behavioural benefits and arousal modulation by temporal expectations in continuous performance tasks, we aimed to replicate our effects with a different task design. We used a task that required discrimination of masked targets appearing either in a fixed rhythm or at variable intervals.

The task parameters were designed to allow modelling of the behavioural benefits of predictability using the Theory of Visual Attention (TVA) (Bundesen, 1990). The TVA provides a mathematical formalisation of the Biased Competition Model of attention (Desimone & Duncan, 1995). In recent years, the TVA has been used to measure how temporal expectations benefit behavioural performance in experimental designs with short, discrete trials (e.g., Sørensen, Vangkilde, & Bundesen, 2015; Vangkilde, Petersen, & Bundesen, 2013). In discrete-trial tasks, temporal expectations induced by hazard rates have been reported to improve Processing Speed selectively (Petersen, Petersen, Bundesen, Vangkilde, & Habekost, 2017; Sørensen et al., 2015; Vangkilde et al., 2013; Vangkilde, Coull, & Bundesen, 2012). We tested whether these findings extended to a continuous-performance context. The selective benefit of temporal structures on processing speed (rather than perceptual threshold) is supported by other tasks using different type of modelling (Drift Diffusion Model; See Cravo, Rohenkohl, Wyart, & Nobre, 2013; Rohenkohl, Cravo, Wyart, & Nobre, 2012).

## 4 Experiment 2

### 4.1 Methods

The experimental procedure was reviewed and approved by the central university research ethics committee of the University of Oxford.

#### 4.1.1 Participants

Participants in this experiment were 30 naïve volunteers (20 of whom were female, mean age 24.5, SD = 4.18). They were recruited through an online research-participation system at the University of Oxford. All had normal or corrected eyesight. Five were left-handed and the rest were right-handed (based on self-reports). They were compensated for their time (£10 per hour).

#### 4.1.2 Apparatus

A PC with an i7 processor and a 2-GB video card was used for displaying stimuli and recording behavioural data. The task was generated using Presentation software (Neurobehavioural Systems, Albany, CA). The stimuli were presented on a 24” LED monitor, with a screen resolution of 1080 × 1920 and a refresh rate of 100 Hz. All stimuli were preloaded to memory using the presentation software to guarantee minimal temporal noise. A video-based eye-tracker (EyeLink 1000, SR Research, Ontario, Canada) was used to measure pupil diameter as well as to monitor eye movements and blinks at 1000 Hz. The recorded data were saved to an eye-tracking PC.

#### 4.1.3 Stimuli

**Fig. 3:**
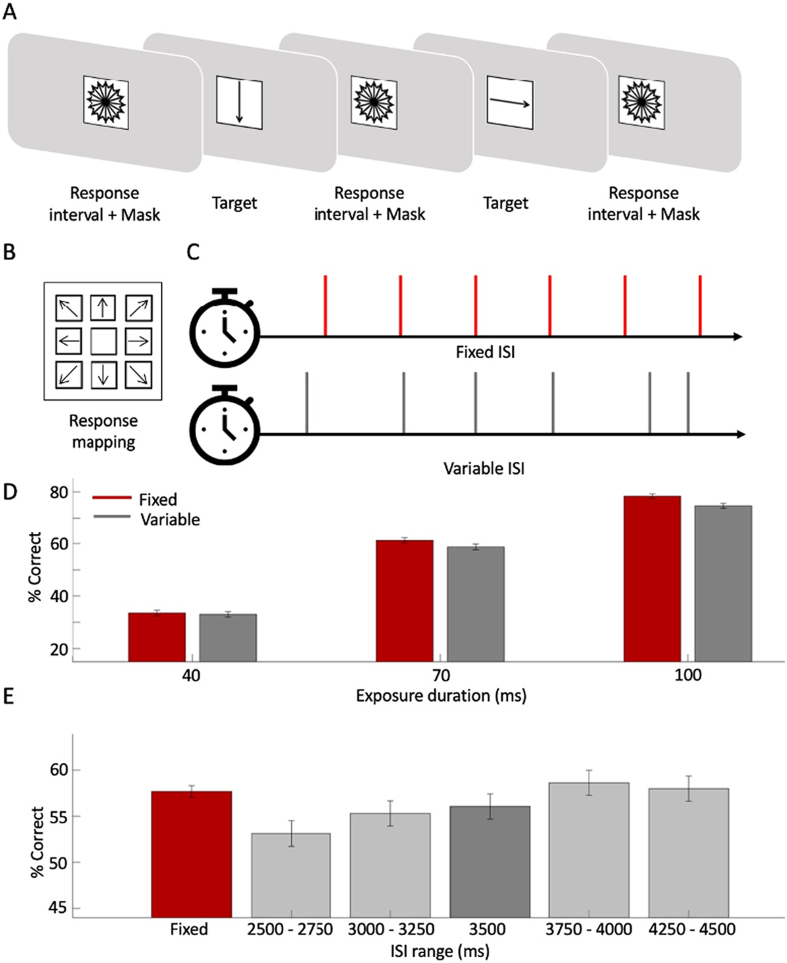
(A) A schematic outline of the experimental design and the response mapping: a visual mask, made of overlapping arrows organized in a circle, appeared at the centre of the screen during the inter-stimulus interval. The inter-stimulus interval was determined according to the experimental condition (with a minimum of 2.5 s); the mask was replaced by a single arrow, appearing for a variable duration: 10/20/40/70/100 ms, and immediately masked again, and participants were instructed to respond while the mask was presented, or do nothing if they did not perceive the target. (B) Participants used the keyboard numpad, where eight arrows were drawn on stickers to indicate the response mapping. As in the illustration, the direction of the arrows corresponded to their locations to assist participants and allow them to respond without moving their eyes from the screen. Participants were instructed to respond only if they were fairly certain to have perceived the stimulus. (C) There were two blocked conditions: a fixed block, in which targets appeared in a fixed 3500-ms rhythm (red) and a Variable block in which intervals varied between 2500 and 4500 ms based on a flat distribution of 20 possible intervals equally spaced every 100 ms (D) comparing percentage of correct responses to targets appearing after 3500 ms intervals in the two experimental conditions (Fixed – in red and Variable – in Grey), by exposure duration. Error bars represent the standard error of the mean. (E) The red bar represents overall performance in Fixed blocks, during which all intervals were equal to 3500 ms. The grey bars depict the mean performance on different subsets trials in variable blocks, based on the inter-stimulus intervals. Error bars represent the standard error of the mean. (For interpretation of the references to colour in this figure legend, the reader is referred to the web version of this article.)

Participants in Experiment 2 performed a continuous task in which they had to make perceptual discriminations based on stimuli that briefly interrupted a mask at regular (3500 ms, Fixed condition) or variable (2500–4500 ms, Variable condition) intervals. In order to apply TVA to test the nature of benefits conferred by temporal expectation in this CPT, the task required a finer perceptual discrimination on stimuli that varied in duration across trials. Participants were asked to report the direction of arrow stimuli that occasionally interrupted a mask stimulus during extended task blocks (see Fig. 3a for a schematic). The mask was a black stimulus comprising 16 bi-directional black arrows circumscribed in a black circle appearing at the centre of the screen (see Fig. 3a). The total size of the mask was approximately 4.5° of visual angle horizontally and vertically. Following the presentation of the mask, a single target arrow pointing at one of eight possible directions (in a square occupying 4.5°) appeared for a varying duration (10/20/40/70/100 ms) and was immediately replaced by the mask. Participants were instructed to try to identify the target arrow and to indicate its direction using the arrow numpad, in which eight arrows pointing at different directions appeared at a corresponding location (see Fig. 3a). Responses were recorded during the successive inter-stimulus interval, during which the mask was presented.

The task consisted of 8 blocks with 100 trials. Each block lasted approximately 6 min. The blocks alternated between two conditions, which varied according to temporal expectation. In the Fixed blocks, a target appeared predictably every 3500 ms. In Variable blocks, the target appeared unpredictably at intervals between 2500 and 4500 ms (mean 3500 ms) with an equal probability for each ISI (drawn from nine possible intervals, equally spaced between 2500 ms and 4500 ms). Fixed and Variable blocks alternated. Half the participants commenced with a Fixed block, and the other half with a Variable block.

We estimated how well participants identified ‘targets’ (arrow figures) that appeared at the centre of the screen every few seconds, and compared performance when the onset of the targets was temporally predictable vs. unpredictable using a computational model based on the theory of visual attention (Bundesen, 1990; Dyrholm, Kyllingsbæk, Espeseth, & Bundesen, 2011). For comparison to other standard tasks, we also compared simple accuracy measures for visible targets (40, 70, and 100-ms exposure) between conditions.

#### 4.1.4 Procedure

The experiment was conducted in a dark testing room. Participants sat 50 cm from the monitor, and a chin rest was used to keep their head still. The eye-tracking device was placed near the monitor and was set to record the two eyes by default. The session began with a short procedure of calibrating the eye tracker. The task instructions then appeared on the screen and were explained to the participant by the experimenter. The participants were told that a visual mask would appear at the centre of the screen, to be replaced briefly every few seconds by a single arrow pointing in one of eight different directions, corresponding to the arrows on the numpad. They were also told that the arrow would be replaced immediately by a visual mask. Whenever the mask appeared, they were to indicate the direction in which the target arrow pointed.

Participants were asked to be as accurate as possible and to keep their eyes fixed on the mask while responding. The analogous spatial organisation of the response mapping on the numpad made it possible to respond while maintaining fixation. Instructions followed suggested TVA recommendations (Sørensen et al., 2015). It was emphasised that the speed of responses was not important. Participants were told that if they did not perceive any arrow, they could simply skip the trial. However, they were encouraged to guess if they were ‘fairly certain’ of having identified the arrow. At the end of each experimental block, participants received feedback indicating their accuracy level over the block. Accuracy was calculated based only on the responses provided, ignoring skipped trials. Participants were asked to aim for 80%–90% accuracy and thus encouraged to guess less if their accuracy fell below 80% and to guess more if performance exceeded 90% (see Sørensen et al., 2015).

Before beginning the experimental session, a short practice session with variable inter-stimulus intervals (2500–4500 ms) consisting of 20 trials allowed participants to learn the task. During the practice block, the first 10 trials presented the targets for an extended duration of 120 ms. The experimenter monitored the participants' responses at this stage to ensure they understood and followed instructions. The experiment itself consisted of eight blocks (each experimental block lasting approximately 6 min). Each block was followed by a short break (approximately one minute). In each block there were 100 trials. There were four Fixed blocks and four Variable blocks. In all, there were 800 experimental trials (400 for each condition) excluding practice.

#### 4.1.5 Statistical analysis

##### 4.1.5.1 Behavioural data

We tested whether temporal predictions influenced performance parameters in a continuous-performance task using TVA modelling parameters and simple accuracy measures.

For the modelling analysis, the data extraction and fitting procedures were performed using Matlab (Ver. 2019a; MathWorks, Inc., Natick, MA) and the LibTVA (Dyrholm et al., 2011; Kyllingsbæk, 2006). The behavioural dataset was first split according to experimental condition: Fixed (4 blocks) or Variable (4 blocks). The calculation of the theoretical attentional parameters (processing speed and perceptual threshold) was based on a maximum-likelihood fitting procedure introduced by Kyllingsbæk (2006) to model the observations based on the TVA framework, using the default modelling parameters (K model ‘free’; exponential fitting). The fitting algorithm output includes two theoretical parameters: (1) Parameter *t*_*0*_ is the perceptual threshold, defined as the longest exposure duration that does not evoke conscious perception, measured in seconds; (2) Parameter *v* is the visual processing speed, or processing rate, measured in the number of target items processed per second. We then compared the resulting parameters between the two conditions.

For the analysis of the unmodelled empirical data, we compared the accuracy rate between conditions for targets presented for 40, 70 and 100 ms. We focused on these exposure durations, as eight participants had missing valid responses in shorter durations. We used a Generalised Linear Mixed-Effect (GLME) approach for the current analysis, as it is more reliable when different conditions may have different trial numbers (Baayen, Davidson, & Bates, 2008). In our current design, different exposure duration led to significantly different numbers of valid trials.

Using the GLME we compared target identification in two conditions (comparable to our contrast in Experiment 1) while also accounting for the additive effect of exposure duration on target detection. Thus, the model included the effects of Block Type (Variable vs. Fixed) and Exposure Duration (40, 70, and 100 ms) as fixed factors, and a random-effects structure that included intercepts for each participant, as well as by-participant slopes for Block Type and Exposure Duration.

##### 4.1.5.2 Pupillometry data

We repeated the same procedure as in Experiment 1. When running the regression model to predict accuracy using pupil size and rhythmic structures, we also added the exposure duration as another predicting factor. For consistency with our behavioural results, we focused on performance that followed stimuli with 40-, 70-, and 100-ms exposure durations.

##### 4.1.5.3 Data exclusion

We applied the same criteria for data exclusion as in Experiment 1. Four participants had a substantial number of missing trials (50% and more) and were excluded prior to any further analysis. The final set of analyses included 26 participants.

### 4.2 Results

#### 4.2.1 Behavioural data

Data were modelled separately for each block type (Fixed vs. Variable) using the LibTVA (Dyrholm et al., 2011; Kyllingsbæk, 2006), a MATLAB toolbox available online to generate TVA model based on empirical datasets. The average maximum likelihood estimation was −176.24 (SD = 38). To validate the reliability of the model, we calculated the correlations between the original data and the predicted data on each exposure duration. The mean Pearson's r score between the predicted and the original performance data was 0.95, confirming a high goodness of fit. The processing-speed parameter was affected by temporal expectations, with faster processing rate in Fixed (*v* = 17_items/__min_; SE = 1.6) compared to Variable (*v* = 15_items/__min_; SE = 1.4) blocks; t(25) = 2.18; *p* < .05; 95%CI[0.08;3.09]). There was no difference in the perceptual threshold between the two conditions (t(25) = −1.631; *p* = .115; 95%CI[−2.83; 0.32]).

Comparing the rate of correct responses for targets presented for visible targets (40, 70, or 100 ms; see Methods section) provided a complementary measure of the benefits of temporal expectation on performance. To compare performance in Fixed and Variable blocks, we focused on the common 3500 ms interval (see Fig. 3b). The results indicated a significant difference in accuracy between conditions (Coefficient Estimate = 0.09; SE = 0.04; t(7490) = 2.22; *p* = .025; 95%CI = [0.01;0.16]). Participants were more accurate when responding to targets presented at a fixed rhythm (0.58; SE = 0.005) compared to variable rhythm (0.56; SE = 0.013). There was also a main effect of exposure duration on accuracy (t(7490) = 14.52; *p* < .001; 95%CI = [0.03;0.04]), but the two variables did not interact (*p* = .6).

As in Experiment 1, the data in the Variable blocks also revealed benefits in performance that followed the increasing passage of time and hazard rate (Fig. 3c). To evaluate this pattern statistically, we modelled the data using a GLMM with the ISI range as a fixed factor, and the mean accuracy as the dependant variable (see Methods section for full model specifications). We included only trials in Variable blocks. The result indicated a significant positive coefficient (0.084; SE = 0.025) (t(4450) = 3.29; *p* = .001; 95%CI = [0.03;0.13]).

#### 4.2.2 Pupillometry data

**Fig. 4:**
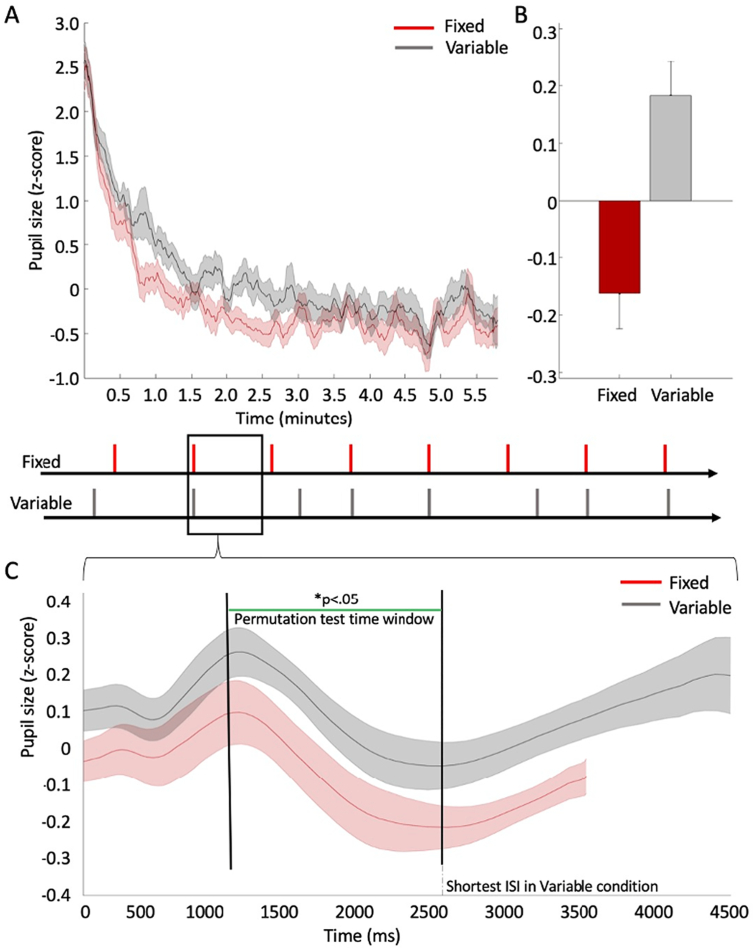
(A) average pupil size (standardised) in Fixed blocks (in red) and Variable blocks (in grey) over the duration of experimental blocks, across all participants, lasting approximately 6 min. Lines represent the mean and shaded error bars represent standard error. For illustration purposes, data were smoothed using a time window of 7000 ms (equivalent to an average duration of two trials). For analysis, the data were standardised by converting the raw pupil size to z scores. The conversion was done separately for each participant, based on the mean pupil size (and standard deviation) throughout the entire experimental run (irrespective of experimental condition). (B) Comparison of the mean pupil size (standardised z-scores) between Fixed blocks vs. Variable blocks. Error bars represent standard error of the mean. (C) Average pupil size (standardised) in Fixed blocks and Variable blocks over the duration of experimental trials, across all participants. Lines represent the mean, and shaded error bars represent the 95% confidence interval range. All trials in Fixed blocks lasted 3500 ms. In Variable blocks, trial duration varied between 2500 and 4500 ms. Therefore, the grey time series includes a varying number of observations within the range of 2500–4500 ms following stimulus offset. For illustration purposes, data were smoothed using a time window of 250 ms. For analysis, the data were standardised by converting the raw pupil size to z scores. The conversion was done separately for each participant, based on the mean pupil size (and standard deviation) throughout the entire experimental run (irrespective of experimental condition). We used a permutation t-test to compare the time series within the range of 1000 to 2500 ms following stimulus offset. The permutation t-test was based on 50,000 samples. The resulting distribution for each data point (each millisecond of recorded data) was compared to a critical t-value (p < .05) adjusted for multiple comparisons based on the ‘t-max’ method (Blair & Karniski, 1993). The green line indicates the time points at which the two time-series differed significantly.

To compare tonic modulation of pupil size between Fixed and Variable blocks, we contrasted the mean pupil size over the full duration of the two different block types. Fig. 4a shows the average standardised pupil size throughout entire experimental blocks, separated by block type. The two standardised signals were compared using a permutation *t*-test with the mean standardised pupil size across the entire block on each condition as the dependent variable (see Fig. 4b). There was a significant difference (t(25) = −2.94; *p* = .007; 95%CI[−0.58; −0.10]), indicating tonically larger pupil sizes in the Variable condition compared to the Fixed condition.

As in Experiment 1, our second set of analyses focused on trial-wise pupil modulation. We constructed time series of the standardised pupil size immediately after stimulus offset and throughout a trial until the onset of the subsequent stimulus, to observe event-locked changes in pupil size. Fig. 4c shows the contrast between the mean pupil size in Fixed blocks and in Variable blocks. After the minimal inter-stimulus interval in Variable blocks (2500 ms), time series include a varying and decreasing number of observations until the maximum interval of 4500 ms.

We used the stimulus-locked time series to retest for tonic differences, this time at a trial level. The approach followed that used for Experiment 1. We used a permutation *t*-test comparing pupil size after the initial stimulus-evoked response had settled (1000 to 2500 ms) and confirmed a consistent difference between the conditions throughout the time window of interest, as indicated by a green line () Fig. 4c.

As in Experiment 1, a gradual increase in pupil size occurred after the initial stimulus-evoked response in both Fixed and Variable conditions until the onset of the next target. To test for significant increases in pupil size in the Variable condition, we used a linear model to estimate the slope of increase, repeating the same procedure as in Experiment 1: for each participant, we calculated the mean time series of pupil size between 2000 ms after onset, until 4500 ms. The data were then fitted and compared to a constant model. The result indicated a significant positive slope (R^2^ = 0.199; F(1, 64,998) = 16,156; *p* < .001; partial η^2^ = 0.199). We also calculated the linear correlation between the time series of pupil size and the Cumulative Distribution Function of the interstimulus intervals based on the experimental data to test the relationship between hazard rate and pupil size directly. The results indicated a significant correlation with a medium effect size (r(64998) = 0.44; p < .001).

Finally, we modelled accuracy data using a single model accounting for pupil size and temporal regularities. There was a significant, negative linear association between the linearly fitted standardised pupil size and accuracy (β = −6.64; SE = 2.75; z = −2.41; *p* = .015). There were no other significant effects (all p's > 0.28).

## 5 General discussion

Across our two experiments, we found clear evidence that temporal structures embedded in continuous-performance tasks benefit behaviour and modulate both tonic and short-scale pupil dynamics, pointing to efficient regulation of arousal at multiple timescales. When fixed temporal intervals maximised temporal expectations, superior behavioural performance was achieved while a more efficient energetic state was maintained overall. When variable intervals were used, arousal levels were elevated overall but were also sensitive to the passage of time within trials, possibly reflecting conditional probabilities learned over the trial history of the experimental block.

Using pupil size as a proxy measure of arousal, our results revealed striking and reproducible effects of temporal regularities on the arousal function, thus revealing arousal to be flexible and adaptive – adjusting to regularities at different time scales. Within CPT, we observed the tonic level of arousal to adjust in line with the level of temporal uncertainly. The continuous context of our task, in which individuals cannot temporarily disengage between discrete trials, was especially conducive for observing the changing patterns of arousal. In Fixed blocks, with high temporal certainty, arousal levels were lower overall. In Variable blocks, with relatively higher temporal uncertainty, participants maintained tonically elevated levels of arousal. Pupil dilation also seemed sensitive to local modulations of arousal, following temporal prediction or temporal preparation when intervals were variable. In the context of Variable blocks, we observed transient increases in pupil dilation that followed the passage of time and the mounting temporal conditional probability for the occurrence of relevant event as the trial interval increased between 2500 and 4500 ms.

Interestingly, while our experimental manipulation revealed a direct link between temporal expectations and arousal, as well as temporal expectations and performance – the relationship between pupil size and behaviour was not straightforward. When looking at pupil dynamics in both conditions alongside performance patterns, the relationships seem contingent on the temporal context. For example, performance was highest in a fixed context, when overall pupil size (as a proxy of arousal) was relatively small in comparison the variable condition. However, within a variable context, our data indicates potentially different relationships between pupil size and performance: both performance and pupil diameter increase as a function of time. Target predictability led to better performance alongside arousal reduction. A unified regression model examining performance accuracy confirmed this association. In both experiments, there was a negative association between pupil size and accuracy. In Experiment 1, a significant interaction with predictability further revealed that pupil size and accuracy were significantly related only when stimuli were presented in a fixed rhythm. Notably, the two experimental designs differed in their behaviour aims – which may explain, in part, differential patterns (Shalev, Nobre, & van Ede, 2019; Shalev & van Ede, 2021). Other meaningful factors likely to have influenced arousal across experiments were the presence of short breaks between blocks in Experiment 2 and substantial differences in length, response mapping and complexity. However, overall, our data suggest that the downregulation of arousal is beneficial for behavioural performance in extended contexts.

Our experimental design was not optimised to look at trial-wise correlations, due to the relatively small number of trials within each experimental condition (if considering the interstimulus interval and exposure duration factors, as well as the varying visual stimuli). The overall pattern we observe is suggestive of different “modes” of performance when varying the temporal context. This resembles other works on “rhythmic” vs. “random” modes (Schroeder, Herrero, & Haegens, 2014), albeit within a different time scale of seconds rather than milliseconds.

Our findings align well with the framework of the Adaptive Gain Theory, which proposes that an increase in uncertainty leads to tonic increases in arousal (Aston-Jones & Cohen, 2005). In the context of reinforcement learning, high levels of uncertainty have been linked to a state of ‘exploration’ and continuous high tonic arousal, whereas reduced uncertainty promotes ‘exploitation’ and reduced tonic arousal (Angela and Dayan, 2005). Whereas previous studies have reported pupillary changes related to un/certainty of stimulus identity and their reward associations (e.g., Friedman et al., 1973; Lavín, San Martín, & Rosales Jubal, 2014; Preuschoff, 't Hart, & Einhauser, 2011; Urai et al., 2017; Vincent et al., 2019), our study extends this notion in sustained-attention context and CPT.

A somewhat more refined theoretical distinction that is highly relevant for our study has been made between expected and unexpected uncertainty in the context of arousal. According to Angela and Dayan (2005), there are different forms of uncertainties in our environment that are differentially encoded by the neuromodulators noradrenaline and acetylcholine. According to their framework, noradrenaline (which is linked with pupil response) primarily responds to uncertainty that cannot be anticipated (i.e., not knowing that the upcoming events cannot be predicted). This contrasts with situations in which we know to predict and prepare for uncertainty. In our task, such unexpected uncertainty emerges when switching from fixed to variable temporal contexts in a way that may have contributed to differences in arousal. However, our results are unlikely to be solely attributable to unexpected uncertainty between blocks. Tonic differences extended well beyond the first trial and therefore were not simply the consequence of encountering an unexpected event at the boundary between conditions.

In both Fixed and Variable CPT conditions, there were temporal structures available to aid performance. However, in the Fixed condition these were more robust: temporal anticipation could rely on a high degree of predictability. The high temporal certainty in the Fixed blocks afforded a state of exploitation in which tonic arousal remained low. Participants could anticipate the task-relevant events, and regulate arousal only transiently, to perform at high levels. In contrast, variable blocks required a higher degree of temporal exploration, in which participants ‘forage’ for task-relevant events over time.

In line with previous studies, we were able to reveal changes in pupil size occurring proactively in the period of anticipation before stimulus onset (Friedman et al., 1973; Jennings, Van Der Molen, & Steinhauer, 1998). However, to our knowledge, our study is the first to show the overall adaptation of arousal operating mode within continuous-performance tasks. These measures of arousal modulation can be directly linked to temporal expectation, and isolated from motor decisions and reward.

Our behavioural-performance results also provide an important advancement to previous work by showing that temporal expectation improves perceptual capacity within extended task contexts. Although it was previously shown that individuals benefit from temporal anticipation based on rhythmic structures in a continuous task (e.g., Dankner et al., 2017), changes to perceptual accuracy was not measured and behavioural outcomes were restricted to response speed. To our knowledge, we present the first evidence for perceptual benefits of temporal expectation in CPT independent of motor preparation. Participants were unable to prepare a specific motor response in advance and thus it was possible to isolate temporal expectations from motor selection or preparation. However, a mounting anticipation of having to respond generally may still have occurred and energising action systems may indeed be an intrinsic aspect of temporal expectation (Nobre, 2001). Importantly, our tasks placed no emphasis on motor performance: In Experiment 1, we used a task that yielded individual differences in perceptual parameters by using forward and backward masking (Shalev, Humphreys and Demeyere, 2016, Shalev, Humphreys and Demeyere, 2018; Shalev, De Wandel, Dockree, Demeyere, & Chechlacz, 2017; Shalev, Vangkilde, et al., 2019). In Experiment 2, participants performed fine perceptual discriminations. They were encouraged to focus on accuracy rather than speed and were permitted to skip responses. By manipulating stimulus durations and modelling the results, we replicated the benefit of temporal expectation for visual processing speed previously noted in single-trial tasks (Sørensen et al., 2015; Vangkilde et al., 2012; Vangkilde et al., 2013) in a continuous-performance context. In line with how instructions and feedback during our TVA task tightly controlled performance accuracy, effect sizes were relatively small when comparing accuracy in fixed and variable temporal contexts. As common practise to support TVA modelling (e.g., (Sørensen et al., 2015; Vangkilde et al., 2012; Vangkilde et al., 2013), participants were encouraged to maintain accuracy within a fixed range (80%–90%), thereby reducing variability in performance measures.

From a wider perspective, it is important to appreciate that rhythmic CPTs are widely used in basic and clinical research and applications, often employed as an elementary neuropsychological tool when assessing sustained attention. Interestingly, a recent study showed impaired capacity to form temporal predictions on a CPT among individuals diagnosed with ADHD (Dankner et al., 2017). Our findings may therefore carry a further implication: the inability to form temporal predictions may also reflect an inability to make the appropriate adjustments of arousal. Such a hypothesis provides an important link between the potential timing difficulties in ADHD and the capacity of sustaining performance over extended time. Indeed, a significant proportion of popular tasks for cognitive assessment of attention difficulties relies on CPT designs with a fixed rhythmic structure (e.g., Conners & Staff, 2000; Lee & Park, 2006; Robertson, Manly, Andrade, Baddeley, & Yiend, 1997). Based on our findings, future inquiries may wish to study sustained performance not only using fixed, but also variable intervals, to understand the interactions between arousal and anticipation among diverse populations.

## Author contributions

NS and ACN developed the study concept and wrote the manuscript. NS programmed the experiment, collected, and analysed the data.

The experimental data used in the paper can be found in the OSF repository: https://osf.io/ch6au/.

## Declaration of Competing Interest

None.
